# Health-related quality of life in COVID-19 in the United Kingdom: a vignette study

**DOI:** 10.1186/s13561-026-00781-5

**Published:** 2026-05-20

**Authors:** Dionysios Ntais, Viola Ntim, Samantha Barton, Alex Porteous, Alvin Ng, Jennifer Page, Hardik Goswami, Victoria Coles, Amy Puenpatom

**Affiliations:** 1 Merck Sharp & Dohme (UK) Limited, London, UK; 2 Costello Medical, London, UK; 3 Costello Medical, Singapore, Singapore; 4 Costello Medical, Manchester, United Kingdom; 5 Merck & Co., Inc., Rahway, New Jersey USA

**Keywords:** COVID-19, Health related quality of life, EQ-5D, Vignette study

## Abstract

**Background:**

Coronavirus disease 2019 (COVID-19) is an acute respiratory illness caused by the severe acute respiratory syndrome coronavirus 2 (SARS-CoV-2). Severe disease may require hospitalisation and respiratory support, which can cause longer-term morbidity. Patient utility data are limited, especially for severe disease where data collection can be challenging or unethical. A vignette study was designed to estimate utility values for COVID-19 disease states.

**Methods:**

A sample of United Kingdom (UK)-based adults (*N* = 500) completed the EQ-5D-5 L questionnaire for eight vignettes as patient proxies in September 2021. Vignettes qualitatively described hospitalisation and disease severity permutations, informed by a large UK-based COVID-19 infection survey. Recruitment was stratified to reflect UK demographics. EQ-5D utilities were derived using the appropriate UK value set.

**Results:**

Recovery without long-term sequelae had the highest utility (mean [standard deviation]: 0.87 [0.14]), followed by pre-infection (0.73 [0.22]). The intensive care unit state had the lowest utility (–0.38 [0.14]). Mild and moderate outpatient states reported similar utility (0.29 [0.26] vs. 0.31 [0.27]). The severe general hospital ward state had lower utility than the high dependency unit state (–0.18 [0.24] vs. − 0.11 [0.25]). Recovery with long-term sequelae had low utility (0.21 [0.29]).

**Conclusions:**

Mean utility values declined as COVID-19 disease state severity increased, suggesting that COVID-19 is likely to impact patient quality of life, in line with published literature. However, the evolving nature of COVID-19 may limit the generalisability of findings to the current disease landscape. Nevertheless, generation of utility values using a vignette-based approach represents a reasonable alternative where collection patient health-related quality of life data is infeasible, albeit with some limitations.

**Trial registration:**

Not applicable.

**Supplementary Information:**

The online version contains supplementary material available at 10.1186/s13561-026-00781-5.

## Introduction

Coronavirus disease 2019 (COVID-19) is an acute respiratory illness caused by the novel severe acute respiratory syndrome coronavirus 2 (SARS-CoV-2) that has resulted in considerable global burden on health care systems and significant mortality [1, 2]. As of 31st March 2024, over 775 million cases of COVID-19 have been reported to the World Health Organization (WHO) worldwide [2]. Over 1.1 million people with COVID-19 in the United Kingdom (UK) have been admitted to hospital, and approximately 232,000 deaths have been recorded [2, 3]. Since May 2023, COVID-19 is no longer considered a Public Health Emergency of International Concern by the WHO [4]. Nevertheless, COVID-19 remains a challenge, with 14,671 admissions to hospital with COVID-19 and 37,750 COVID-19 diagnoses in hospital in England in 2025 [5]. 

COVID-19 is known to cause a wide spectrum of symptoms, ranging from mild symptoms of the upper respiratory tract to life-threatening sepsis; [6] treatment and management options for COVID-19 are dependent on disease severity. Severe disease may require hospitalisation and respiratory support, which can lead to longer-term morbidity [7]. Patients with comorbidities such as diabetes, cardiovascular conditions or chronic obstructive pulmonary disease (COPD) are at increased risk of progression to severe disease [8]. 

There is a growing body of evidence suggesting that COVID-19 can detrimentally impact patients’ health-related quality of life (HRQoL), both physically and psychologically, with the impact persisting beyond the acute infection phase and with magnitude of impact dependent on factors such as age, comorbidity and severity of illness [9–12]. Previous studies assessing HRQoL in people with COVID-19 have used generic measures such as the 36-item Short Form Health Survey and the EQ-5D questionnaire, disease-specific measures for other pulmonary conditions such as COPD, and, more recently, COVID-19-specific questionnaires such as the COV19-QoL [9, 13]. Whilst disease-specific measures may be more sensitive to specific features of the disease, generic measures are favoured by Health Technology Assessment (HTA) bodies to inform health state utility values (HSUVs) in economic models, facilitating comparability across conditions.

The most established generic measure of HRQoL is the validated EQ-5D questionnaire, which is recommended by the National Institute for Health and Care Excellence (NICE) to derive HSUVs, referred to hereinafter as “utility data” [14]. 

Accurate and comprehensive utility data for COVID-19 are essential to inform economic models assessing the cost-effectiveness of new health technologies. However, within published literature, HRQoL data among people with severe COVID-19 are limited. People with severe COVID-19 may be hospitalised and/or unconscious, and, as such, collection of patient-completed HRQoL measures can be challenging and unethical.

In an previous NICE appraisal of therapies for COVID-19 (TA878), stakeholders criticised the use of data from a non-COVID-19 population to inform utility decrements for severe COVID-19 [15]. The use of COVID-19 severity-specific vignettes with EQ-5D questionnaires completed by the UK general population was proposed, in line with NICE recommendations [14, 15]. Vignettes can be used to describe different hypothetical situations to collect quantitative or qualitative insights. When patient-completed HRQoL measures are not feasible or appropriate, a vignette-based approach can supplement important data gaps for decision-making. Vignette studies have informed economic evaluations across various HTA bodies, including NICE, for example, in rare diseases where elicitation of HRQoL is challenging due to small patient numbers [16]. 

A large UK-based vignette study was conducted to estimate HRQoL utility values for non-death COVID-19 disease states, addressing gaps where generation of patient-reported utility values is infeasible.

## Methods

### Study design

A vignette-based approach was used to describe eight hypothetical COVID-19 infection permutations with varying disease severities and associated hospitalisation status in a qualitative framework. Using SurveyMonkey (Survey-Monkey Inc., San Mateo, California, USA; www.surveymonkey.com), five hundred UK adults completed the EQ-5D-5 L, acting as proxies on behalf of people with COVID-19. Participant responses to the EQ-5D-5 L were mapped to EQ-5D utility index scores using the appropriate mapping algorithm and UK value set [14, 17]. 

### Participants

Members of the UK general public were recruited in September 2021 via Prolific (www.prolific.co), an online panel of people willing to undertake research surveys. A small financial incentive (£3.13) was offered to participants recording complete and high-quality responses; all responses were collected anonymously. UK Office of National Statistics (ONS) general population age, sex, and ethnicity distribution data informed sample stratification, ensuring a demographic distribution reflecting that of the UK population and ensuring generalisability of study results.

The study sample size was selected with reference to previous studies investigating HRQoL after COVID-19 infection [18, 19]. Eligible participants were those aged at least 18 years, living in the UK, and willing and able to give consent to participate. Participants were not required to have had prior COVID-19 infection, although participants’ COVID-19 infection (prior and current) status, vaccination status, and COVID-19 status of close friends or family were collected to facilitate exploratory subgroup analyses.

### Vignette development

Eight vignettes were designed with reference to the recommendations outlined in the report by the NICE Decision Support Unit (DSU) and Matza et al. (2021) [20, 21]. Vignettes described eight hospitalisation and disease severity states for COVID-19: baseline (pre-infection), outpatient (mild), outpatient (moderate), general hospital ward (severe), high dependency unit (HDU; severe), intensive care unit (ICU; critical), recovered (no long-term sequelae), and recovered (long-term sequelae). These disease states reflect the natural progression of COVID-19 over time and were deemed by two company clinical experts specialising in COVID-19 (UK and USA based) to capture the key stages in the disease pathway. These experts provided qualitative input in multiple virtual meetings and reviewed draft survey documents. All vignettes also included “underlying health conditions”, to align more closely with people who would be eligible for COVID-19 antivirals according to MHRA licensed indications. These underlying health conditions were not specified to avoid overcomplicating the design process and impact study requirements. Therefore, several concepts were included in the vignettes to describe attributes of the hypothetical health states: disease severity and treatment setting, signs and symptoms of COVID-19, underlying health conditions and risk factors, ventilation status, and long-term complications (Table 1). Further details and full vignette descriptions are presented in Additional file 1.

**Table 1:** Overview of the vignettes

Disease state	S1	S2	S3	S4	S5	S6	S7	S8
*Description of condition*	No COVID-19	COVID-19	COVID-19	COVID-19	COVID-19 where the patient requires supplemental oxygen through a face mask	COVID-19 where the patient cannot breathe on their own and will die if not treated	Recovered from COVID-19 with no long-term health issues	Recovered from COVID-19 and suffering from long term health issues as a result
*Disease severity*	N/A	Mild	Moderate	Severe	Severe	Critical	N/A	N/A
*Treatment setting*	N/A	Not in hospital	Not in hospital	General hospital ward	High dependency unit in a hospital	Intensive care unit in a hospital	N/A	N/A
*Ventilation status*	N/A	None	None	Via nasal canula	Via face mask	Intubated	N/A	N/A
*Underlying health condition*	Present	Present	Present	Present	Present	Present	Present	Present
*Symptoms*	N/A	Fever	Fever	Fever	Fever	N/A	N/A	N/A
		Cough	Cough	Cough	Cough			
		Fatigue	Fatigue	Fatigue	Fatigue			
		Headache	Headache	Confusion	Confusion			
		Muscle pain	Muscle pain	Muscle pain	Muscle pain			
		Loss of smell	Loss of smell	-	-			
		Nasal congestion	Nasal congestion	-	-			
*Long-term complications*	N/A	N/A	N/A	N/A	N/A	N/A	None	Fatigue
								Shortness of breath
								Muscle and/or joint pain

*Abbreviations: COVID-19* Coronavirus disease 2019, *N/A* Not applicable

Sources used to inform the development of the vignette symptoms included the study sponsor clinical trial protocol and clinical study report [22, 23], an ONS COVID-19 infection survey [24, 25], a prospective observational study by The International Severe Acute Respiratory and Emerging Infection Consortium [26], and a systematic literature review [27]. Subsequently, one medical expert affiliated to the research organisation supporting with the study conduct independent of the outcomes research team which developed the original survey draft, was consulted to ensure vignette descriptions were reflective of the disease states and used patient friendly language.

Vignettes were drafted using lay person language to be suitable for valuation by the general public. A small internal pilot study was conducted to validate the vignette descriptions, whereby five participants unfamiliar with the study methodology provided feedback on the clarity of the vignette descriptions, structure and user-friendliness of the survey. The vignettes were presented to participants in a fixed random order (consistent across participants due to survey platform restrictions) to prevent potential order bias from presenting increasingly severe disease states.

### Outcomes and covariates of interest

Participants completed the proxy 2 version of the EQ-5D-5 L for each of the eight vignettes, responding how they (the participant) thought the patient would rate their own HRQoL, if the patient were able to communicate it. The proxy 2 was preferred to the proxy 1 (where the proxy is asked to rate the patient’s HRQoL in their [the proxy’s] opinion), since participants were rating the HRQoL for hypothetical disease states, rather than actual patients. Participants completed a warm-up exercise to practise the proxy-reporting task before completing the EQ-5D-5 L for the vignettes.

The EQ-5D-5 L descriptive system measures health status for five dimensions (mobility, self-care, usual activities, pain/discomfort and anxiety/depression) [28]. Responses include a 5-point Likert scale (“no”, “slight”, “moderate”, “severe” and “extreme” problems) for each dimension. The combination of these ratings for the five dimensions gives a unique five-digit number which describes the individual’s health state. The 5 L version was preferred to the 3 L version (based on a 3-point Likert scale), since it was designed to have greater sensitivity [29]. 

Participant age, sex and nationality were obtained via Prolific (www.prolific.co); prior COVID-19 status and vaccination status were collected as part of the survey.

### Study procedures

An external expert in the measurement and valuation of HRQoL reviewed and validated the proposed methodology, including the development and validation of the vignette descriptions, and their presentation to participants. For each vignette, each dimension of the EQ-5D-5 L was presented on an individual page (reviewed and approved by EuroQol [Vendor Agreement 160832]); participants could only move onto the next page of the survey following completion of all questions on the current page, thereby minimising missing data. Partially completed surveys were not included in the study.

Informed consent was not sought as this was a non-interventional study and participant data remained anonymous. However, participants were required to confirm consent to proceed with survey participation, acknowledging that since data were anonymised, it was not possible to withdraw consent following survey completion.

Additionally, one fair check (“attention check”) was added to the survey to ensure that sufficient attention was paid to the survey instructions. At the end of a randomly selected vignette, participants were asked to select a specific response option clearly stated in the question. Submissions that failed the attention check were rejected.

### Data handling

To ensure participants’ responses were sensible and of high quality, participants who failed the attention check were excluded (*n* = 9), as were participants with incomplete responses or an unusually short survey time (< 5 min) (*n* = 8), and participants with illogical responses, where higher utility was reported for the most clinically severe (S6) state versus the least severe (S1 and S7) states (*n* = 2). If a response was rejected, a replacement participant was recruited via Prolific, ensuring the sample remained representative.

### Statistical and analytical methods

In line with current NICE guidelines, responses to the EQ-5D-5 L were mapped to utility index scores via the Hernández Alava et al. (2023) algorithm based on the UK EQ-5D-3 L value set [14, 17]. OpenSAFELY patient characteristics (age and sex) were used in the mapping algorithm to reflect the population of people with COVID-19 in the UK [30]. 

Descriptive statistics and boxplots were used to evaluate utility values for each disease state. Descriptive statistics were also produced for participants’ demographic characteristics. As only complete responses were accepted, imputation of missing data was not required.

Subgroup analyses were conducted with descriptive summaries based on stratification by prior COVID-19 infection (Yes/No), COVID-19 status of close friends and family (Yes/No), and vaccination status (Fully Vaccinated/Partially Vaccinated/Not Vaccinated) to assess whether perception of the vignettes differs between strata. The Wilcoxon rank sum and Kruskal-Wallis H tests were conducted for continuous and categorical variables, respectively. Pooling of estimates across disease states was considered where results indicated no meaningful difference in HRQoL, thus potentially forming more clinically applicable groups. 

## Results

### Baseline characteristics

Five hundred participants completed eligible survey responses. The mean age of participants was 44.15 (standard deviation [SD]: 15.5), 48.8% were male, and 80.4% were white (Table 2). Most participants were fully vaccinated (83.8%), 11.8% reported having previously experienced COVID-19, while 67.8% reported prior COVID-19 experience among close friends or family. A small proportion of participants reported current COVID-19 infection (0.6%).

**Table 2:** Summary of participant characteristics

Characteristic	Value (*N* = 500)
Mean age, years (SD)	44.15 (15.5)
Sex, n (%)	
Male	244 (48.8)
Female	256 (51.2)
Ethnicity, n (%)	
White	402 (80.4)
Black	25 (5.0)
Asian	42 (8.4)
Mixed or Other	30 (6.0)
Unknown	1 (0.2)
Prior COVID-19 infection, n (%)	
Yes	59 (11.8)
No	440 (88.0)
Prefer not to say	1 (0.2)
Don’t know	0 (0.0)
Current COVID-19 infection, n (%)	
Yes	3 (0.6)
No	494 (98.8)
Prefer not to say	0 (0.0)
Don’t know	3 (0.6)
Prior COVID-19 infection of close friends or family, n (%)	
Yes	339 (67.8)
No	155 (31.0)
Prefer not to say	1 (0.2)
Don’t know	5 (1.0)
Vaccination status, n (%)	
Fully vaccinated	419 (83.8)
Partially vaccinated	27 (5.4)
Not vaccinated	51 (10.2)
Prefer not to say	3 (0.6)
Don’t know	0 (0.0)

*Abbreviations: COVID-19* Coronavirus disease 2019, *N* Total number of respondents, *n* Subset number of respondents, *SD* Standard deviation

### Utility values

**Fig. 1:**
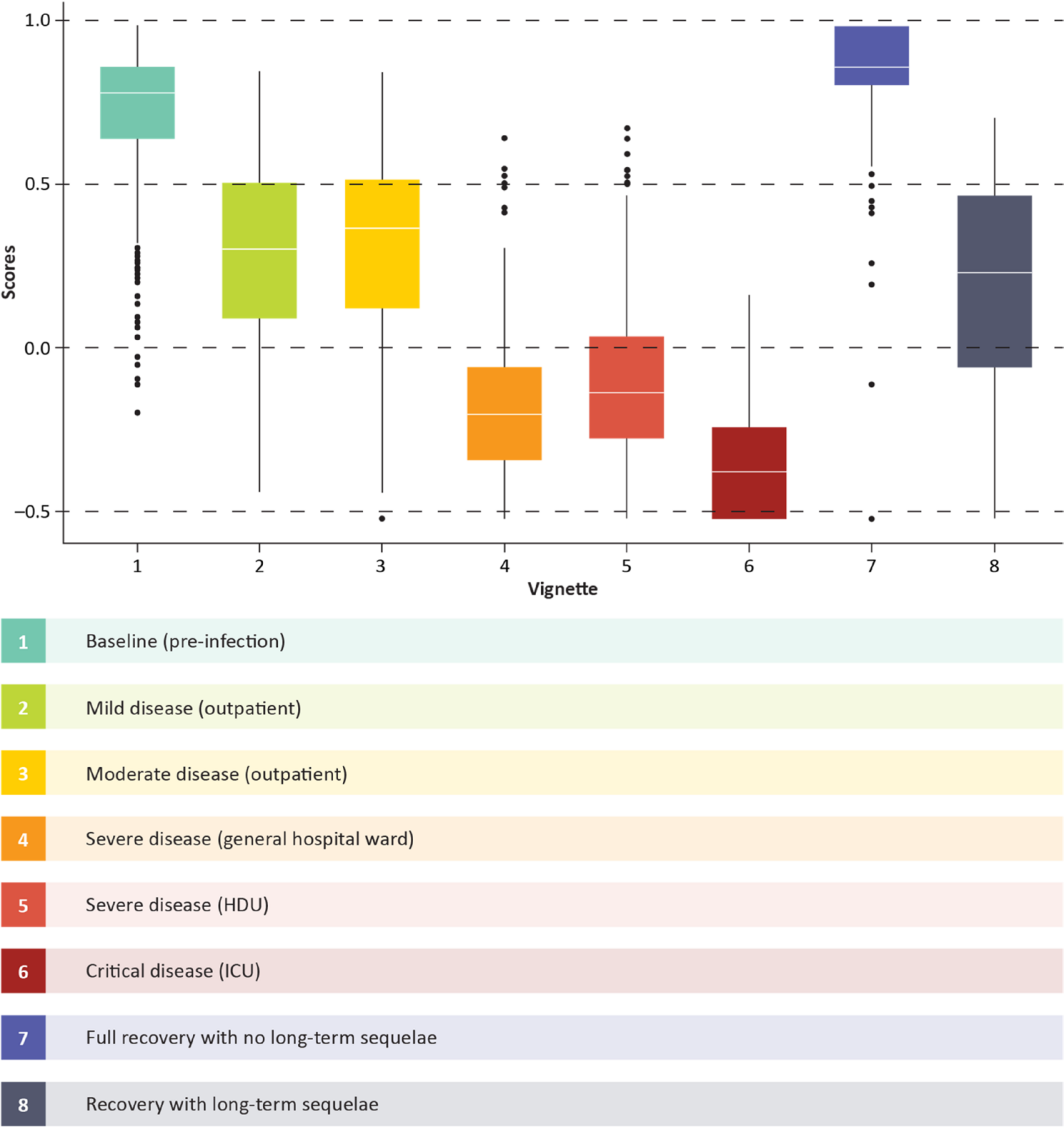
Boxplots for utility values Footnotes: Boxplots represent the median utility value and IQR for each health state. *Abbreviations*: *HDU*, high dependency unit; *ICU*, intensive care unit; *IQR*, interquartile range

As shown in Table 3, the critical ICU state had the lowest utility (S6: ‒0.38 [0.14]) while mild and moderate outpatient states reported similar utility (S2: 0.29 [0.26] vs. S3: 0.31 [0.27]). The severe general hospital ward state had lower utility than the HDU state (S4: ‒0.18 [0.24] vs. S5: ‒0.11 [0.25]). Low utility was also reported for the state reflecting recovery with long-term sequelae (S8: 0.21 [0.29]). The highest utility was reported for the state reflecting full recovery with no long-term sequelae (S7: 0.87 [0.14]), followed by baseline utility pre-infection (S1: 0.73 [0.22]). Boxplots displaying these results are presented in Fig. 1.

**Table 3:** Summary of utility values

Disease state	Mean (SD)	Median (IQR)
S1: Baseline (pre-infection)	0.73 (0.22)	0.78 (0.22)
S2: Outpatient (mild)	0.29 (0.26)	0.30 (0.42)
S3: Outpatient (moderate)	0.31 (0.27)	0.37 (0.39)
S2/S3 (pooled): Outpatient (mild/moderate)	0.30 (0.23)	0.33 (0.33)
S4: General hospital ward (severe)	‒0.18 (0.24)	‒0.20 (0.28)
S5: High dependency unit (severe)	‒0.11 (0.25)	‒0.14 (0.31)
S4/S5 (pooled): Non-ICU inpatient	–0.15 (0.21)	–0.17 (0.28)
S6: ICU (critical)	‒0.38 (0.14)	‒0.38 (0.28)
S7: Recovered (no long-term sequelae)	0.87 (0.14)	0.86 (0.18)
S8: Recovered (long-term sequelae)	0.21 (0.29)	0.23 (0.53)

Footnote: Negative values are mean or median scores and are not disutilities

*Abbreviations: ICU* Intensive care unit, *IQR* Interquartile range, *SD* Standard deviation

The considerable overlap in interquartile ranges (IQRs) and counterintuitive ordering of utilities compared to severity for the mild and moderate outpatient disease states (S2 and S3), and for the severe general hospital ward and HDU disease states (S4 and S5), may suggest that participants could not differentiate between these states on the basis of the vignette descriptions. Pooling utility values was therefore explored: the pooled mild/moderate outpatient (S2/S3 pooled) state had a mean utility of 0.30 (0.23) while the non-ICU inpatient (S4/S5 pooled) state reported a mean utility of ‒0.15 (0.21) (Table 3).

Subgroup analyses stratifying participants by prior COVID-19 infection, COVID-19 status of close friends or family, and vaccination status did not identify any statistically significant results between strata (see Additional file 2).

## Discussion

This de novo vignette study facilitated the generation of utility data for severe COVID-19 disease states where prospective HRQoL data collection may otherwise be unsuitable. Non-death disease states were also explored, permitting comparison with patient-reported published utility data.

Vignette-derived utility values in this study are consistently lower than patient-derived utility values for similar disease states. Whilst this vignette study generally reflects a population expected to have a greater disease burden (i.e. symptomatic patients with an underlying health condition associated with increased risk of severe disease), it is unclear to what extent this is the main driver of the trend identified, or whether this is in part due to the vignette methodology.

Despite describing a less severe disease state, the mild outpatient (S2) state in this study was associated with a lower mean utility value than the moderate outpatient (S3) state; however the difference was marginal (0.02) and the IQRs overlapped considerably, indicating that participants were not able to meaningfully distinguish between these states in terms of their impact on HRQoL. This may be expected as the mild and moderate vignette (S2 and S3) descriptions were identical apart from breathing rate and heart rate, the impact of which may be difficult to interpret in terms of changes in EQ-5D-5 L domain scores. Perception of disease states may be more sensitive to other aspects which were consistent across S2 and S3, such as symptoms and treatment setting.

Similarly, the severe general hospital ward (S4) state was associated with a lower utility value than the severe HDU (S5) state, but the IQRs overlapped considerably. As the vignette descriptions for S4 and S5 were identical except for oxygen delivery and treatment setting, participants may not have been able to distinguish between these states. Alternatively, the utility values may reflect differences in the perception of oxygen delivery method (a nasal canula may sound more invasive than a face mask), and setting (a higher level of care described for HDU may be perceived as favourable compared to a general hospital ward). In both cases, the marginal differences suggest that utility values could be pooled across states should these results be used to inform future economic models.

### Mild/moderate COVID-19

There are three comparable published utility studies for acute COVID-19: a retrospective measurement of HRQoL in people (*N* = 406) with mild-to-moderate COVID-19 at different timepoints during their infection (Soare et al. 2024) [31], a cost-utility analysis of the UK PANORAMIC trial involving the completion of questionnaires by community-based adults (*N* = 12,821) at higher risk of severe COVID-19 outcomes with confirmed COVID-19 infection (Png et al. 2024) [32], and a study assessing HRQoL in non-hospitalised people (*N* = 548) with COVID-19 in England (Sandmann et al. 2022) [33]. Each elicited utilities from patients using the EQ-5D-5 L, but all were nonetheless subject to limitations.

All three studies corroborated our finding of reduced HRQoL associated with mild-to-moderate non-hospitalised COVID-19, although none found as substantial a decrement as the vignette study. The mean utility values for both outpatient disease states (S2: 0.29 and S3: 0.31) in this study are comparable to the utility value reported for the acute phase of COVID-19 for respondents in Soare et al. (2024) who required hospitalisation during their infection (0.38 [0.32]), but lower than the utility value reported for non-hospitalised respondents (0.62 [0.35]) [31]. For the “worst day of COVID-19”, Sandmann et al. (2022) report a mean utility of approximately 0.65 (obtained by graph digitisation), with a wide range and IQR approximately 0.48–0.85 [33]. Png et al. (2024) report baseline utility values (within 5 days of COVID-19 symptom onset) of 0.737 [0.002] for patients treated with molnupiravir and 0.738 [0.002] for those receiving usual care [32]. 

Differences in study design and participant characteristics between the studies limit comparability of results and may explain the lower utility values reported in this vignette study. For example, only 57% of non-hospitalised respondents in Soare et al. (2024) had underlying comorbidities putting them at higher risk of severe COVID-19 [31], and almost three quarters (72.45%) of respondents in Sandmann et al. (2022) had no comorbidities at baseline [33]. All vignettes in this study included the presence of underlying health conditions associated with increased risk of severe disease (Table 1); individuals with existing comorbidities or risk factors for severe COVID-19 have significantly lower general HRQoL than those without [31]. Additionally, patients’ experience of symptoms in the published studies may be less severe than those described by the vignettes, which were designed to reflect symptomatic acute disease. For example, a large proportion of participants in Sandmann et al. (2022) did not report key symptoms included in the outpatient state vignettes (S2 and S3); over 70% did not report a fever, and approximately 50% did not report a new cough within the first seven days of COVID-19 infection [33]. In Png et al. (2024), approximately 80% of the trial participants experienced symptoms for ≤ 3 days, and 73% did not report any major symptoms at baseline [32]. Finally, HRQoL for all health states in Soare et al. (2024) and for the “worst day of COVID-19” in Sandmann et al. (2022) was reported retrospectively and may therefore suffer from recall bias [33]. 

### Hospitalised COVID-19

In this study, the lowest mean utility value was estimated for the critical ICU (S6) severity state. Likely due to methodological challenges, alternative utility values for this state do not exist and NICE appraisals to date have simply assumed a value of 0 [34]. 

The retrospective study of Soare et al. (2024) is the only other source of utility values for hospitalised COVID-19, which reported a value of 0.38, substantially higher than that found here [31]. However, the hospitalised respondents represented a small sample size (*N* = 42), and almost a fifth (19%) had no underlying comorbidities or risk factors for severe COVID-19. Furthermore, this study may suffer from sample selection bias and recall bias, given that patients were reporting their HRQoL retrospectively, following recovery.

### Recovery

The highest mean utility value was reported for the state representing recovery with no long-term sequelae (S7), followed by the pre-infection (S1) state. Considerable overlap was observed in the distributions between these two states, but this was not unexpected given similarities in vignette descriptions for S1 and S7 (Table 1). Higher utility for S7 may reflect the assumed relief associated with recovering from COVID-19 and reduced anxiety around contracting the disease compared with pre-infection. This is reflected in a higher proportion of participants reporting moderate to extreme anxiety/depression for S1 compared with S7 (see Additional file 3).

Soare et al. (2024) found a similar trend in utility values between pre-infection and recovered states [31]. Among adults with mild-to-moderate COVID-19 not requiring hospitalisation during their infection, the mean utility value for the pre-COVID-19 state (0.82 [0.25]) was slightly lower than that for the post-COVID-19 state, i.e. after recovering from all COVID-19-related illnesses (0.84 [0.22]) [31]. Notably, the post-COVID-19 utility values among adults requiring or not requiring hospitalisation during mild-to-moderate infection (0.86 [0.17] and 0.84 [0.22], respectively) were closely aligned to the mean utility value for the vignette reflecting recovery with no long-term sequelae (S7) in this study (0.87 [0.14]).

### Long COVID

This vignette study corroborates findings from previously published literature that long-term sequelae substantially negatively impact HRQoL [31, 35]. The state reflecting recovery with long-term sequelae (S8) had lower utility than both the mild and moderate outpatient (S2 and S3) states, which is similar to the trend observed in multivariable analysis for the hospitalised cohort of Soare et al. (2024) (reporting an impact of − 0.147 versus − 0.186 for the ‘acute phase’ versus ‘long COVID’, respectively) [31]. The general public’s anxiety surrounding ‘long COVID’ may contribute to these discrepancies in reported utility values, with a higher proportion of participants in this study reporting severe or extreme anxiety/depression for S8 compared to S2 and S3 (see Additional file 3). However, the utility value for S8 (0.21 [0.29]) is lower than mean utility values reported for ‘long COVID’ from published studies: 0.70 and 0.54 for non-hospitalised and hospitalised cohorts in Soare et al. (2024), 0.51 (*N* = 82; 17 UK-based) in Tak (2023), and 0.49 (UK-based individuals self-reporting long COVID; *N* = 1,495) in Carlile et al. (2023) [31, 35, 36]. Differences in the proportion of individuals with underlying health conditions may contribute to discrepancies in reported utility values between studies.

#### Strengths

Study strengths include the representative UK population sample, design of the vignettes in accordance with best practice guidelines, and survey design elements that minimised missing data, ensuring response quality and limited bias [20, 21]. 

The vignette-based approach facilitated the estimation of utility data for people with severe COVID-19 disease, for whom prospective HRQoL data collection is otherwise unsuitable, thereby resolving this data gap in future economic evaluations including severe COVID-19 disease states.

#### Limitations

The primary limitation of vignette studies such as this is their reliance on hypothetical disease states. HRQoL was estimated by the general public and not measured directly from patients experiencing these disease states, resulting in a considerable uncertainty surrounding the generalisability of calculated utilities to actual utility experienced by patients in clinical practice. Furthermore, as data were collected via an online survey, there may have been some voluntary response bias as all participants were self-selected.

The validity of the utilities depends on the accuracy and level of detail provided in vignette descriptions to contextualise disease states, which cannot portray all aspects of the patient experience. As such, the utilities may underestimate the variability associated with COVID-19. There is also evidence that participants may have struggled to distinguish between similar vignettes or misinterpreted particular aspects of the disease state descriptions. There is no standardisation regarding the content of a disease state description, and the procedures for developing and valuing vignettes vary across studies. However, the vignettes were designed with reference to the recommendations outlined in the report by the NICE DSU and Matza et al. (2021), and data from existing studies were used to inform the disease states, minimising the impact of this limitation [20, 21]. Additionally, a small pilot study was conducted and medical experts provided input to validate the vignette descriptions.

It is conceivable that previous COVID-19 experience may have impacted participant perception of the vignettes. Subgroup analyses found no significant difference in the responses based on prior COVID-19 infection, COVID-19 status among close friends or family, or vaccination status. This may suggest that these variables are unlikely to have influenced the study findings, although sample sizes were small in some cases. Similarly, the term COVID-19 may have been associated with preconceptions, also in turn impacting how participants interpreted the vignettes. However, it was not possible to omit the label “COVID-19” in order to facilitate the subgroup analyses detailed above.

Lastly, given the evolving COVID-19 landscape, participant responses collected in September 2021, during a period of high public concern about COVID-19, may differ from what would be seen were the study conducted within the current endemic COVID-19 setting.

#### Implications for economic modelling and health technology assessment

This study was initiated to address a gap in the evidence on HRQoL in COVID-19, following stakeholder criticism regarding the use of utilities from other disease areas in initial NICE technology appraisals. It was conducted in line with NICE recommendations, using the EQ-5D, NICE’s preferred instrument. As discussed above, the utility values elicited in this study were somewhat lower than in other studies, which may lead to comparatively large QALY gains associated with efficacious prevention and treatment of COVID-19 in those at high risk of progression to severe disease. Given the limited available evidence, and the evolving COVID-19 landscape, any utility values used within HTA submissions are likely to be subject to scrutiny by assessors and require validation by clinical and patient experts. This issue is part of a wider debate around the true value of such technologies in the wake of a highly disruptive pandemic.

## Conclusion

Overall, mean utility values declined as disease state severity increased, and where comparison was feasible, previous literature demonstrated similar trends of reduced utility within the acute phase of COVID-19 infection and during long COVID, despite considerable differences in methodological design [20, 21]. The vignette-based approach employed therefore provides a reasonable alternative to patient-reported COVID-19 utility values. Whilst previous vignette studies often focus on data collection among rare diseases [16], this study highlights the potential for vignette-based approaches to address data gaps across a wider range of diseases where data collection is ethically challenging due to the nature and severity of the disease.

## Supplementary Information

Additional file 1. Health state vignettes. Provides full descriptions of each of the vignettes presented to participants.

Additional file 2. Subgroup analysis results. Presents a table of the EQ-5D-5L utility scores of vignettes by prior COVID-19 infection status, COVID-19 status of close friends or family, and vaccination status.

Additional file 3. EQ-5D-5L responses by domain. Presents a table displaying the proportion of participants reporting each level of the EQ-5D-5L for each domain.

## Data Availability

The datasets generated and analysed during the current study are not publicly available as explicit permission was not sought from participants to share anonymised dataset more widely, but are available from the corresponding author on reasonable request.

## Abbreviations

COPD: Chronic obstructive pulmonary disease
COVID-19: Coronavirus disease 2019
DSU: Decision Support Unit
HDU: High dependency unit
HRQoL: Health-related quality of life
HSUV: Health state utility values
HTA: Health Technology Assessment
ICU: Intensive care unit
IQR: Interquartile range
NICE: National Institute for Health and Care Excellence
ONS: Office of National Statistics
SARS-CoV-2: Severe acute respiratory syndrome coronavirus 2
SD: Standard deviation
UK: United Kingdom
WHO: World Health Organization
