# Supplementary material for: Health-related quality of life in COVID-19 in the United Kingdom: a vignette study
Source: Health Econ Rev. 2026 May 20;16:85. doi: 10.1186/s13561-026-00781-5 (PMC13366983; doi:10.1186/s13561-026-00781-5)
Supplement: Supplementary file 1 — Additional file 1. Health state vignettes. Provides full descriptions of each of the vignettes presented to participants. [file 13561_2026_781_MOESM1_ESM.docx]

Additional file 1

**Health State Vignettes**

**Description S1: Baseline (pre-infection)**

- The patient **does not have COVID-19**.
- The patient has an **underlying health condition** that makes them more likely to get severely ill from COVID-19. You do not need to consider the impact of this underlying health condition on the patient’s health, only that this condition makes the patient more likely to get severely ill from COVID-19.

**Description S2: Outpatient (mild)**

- The patient has **COVID-19**.
- The patient is **not in hospital**.
- The patient is **not using any devices that supply extra oxygen to the lungs**.
- The patient has a **normal breathing rate** and is not out of breath.
- The patient has a **normal heart rate** at rest.
- The patient has a **fever** – their body temperature is over 38°C. They feel hotter than usual (particularly on their chest and back), have chills and are shivery.
- The patient has a **dry cough** and is coughing more than they usually do.
- The patient feels **extremely tired** and finds daily activities (such as walking up the stairs, getting out of bed, daily chores) difficult.
- The patient has a **headache** that is moderately to severely painful, feels ‘pulsing’, ‘pressing’, or ‘stabbing’, occurs across both sides of the head and lasts more than three days.
- The patient’s **muscles are sore**, particularly their shoulders and legs. The area feels sore to the touch and can prevent the patient from carrying out daily tasks without pain.
- The patient has **lost their sense of smell**, they are unable to smell as they usually would, including strongly scented things like coffee or flowers.
- The patient has a **blocked, stuffy, or bunged-up feeling** in their nose.
- The patient has an **underlying health condition** that makes them more likely to get severely ill from COVID-19. You do not need to consider the impact of this underlying health condition on the patient’s health, only that this condition makes the patient more likely to get severely ill from COVID-19.

**Description S3: Outpatient (moderate)**

- The patient has **COVID-19**.
- The patient is **not in hospital**.
- The patient is **not using any devices that supply extra oxygen to the lungs**.
- The patient experiences **shortness of breath on exertion**, for example, when walking up the stairs.
- The patient has a **high heart rate** at rest.
- The patient has a **fever** – their body temperature is over 38°C. They feel hotter than usual (particularly on their chest and back), have chills and are shivery.
- The patient has a **dry cough** and is coughing more than they usually do.
- The patient feels **extremely tired** and finds daily activities (such as walking up the stairs, getting out of bed, daily chores) difficult.
- The patient has a **headache** that is moderately to severely painful, feels ‘pulsing’, ‘pressing’, or ‘stabbing’, occurs across both sides of the head and lasts more than three days.
- The patient’s **muscles are sore**, particularly their shoulders and legs. The area feels sore to the touch and can prevent the patient from carrying out daily tasks without pain.
- The patient has **lost their sense of smell**, they are unable to smell as they usually would, including strongly scented things like coffee or flowers.
- The patient has a **blocked, stuffy, or bunged-up feeling** in their nose.
- The patient has an **underlying health condition** that makes them more likely to get severely ill from COVID-19. You do not need to consider the impact of this underlying health condition on the patient’s health, only that this condition makes the patient more likely to get severely ill from COVID-19.

**Description S4: General hospital ward (severe)**

- The patient has **COVID-19**.
- The patient is **in hospital on a** **general ward**.
- The patient is using a device in which **oxygen is delivered through a tube through the nose**, allowing the air into their lungs.
- The patient is **short of breath at rest**.
- The patient’s **heart rate is very high**.
- The patient has a **fever** – their body temperature is over 38°C. They feel hotter than usual (particularly on their chest and back), have chills and are shivery.
- The patient has a **dry cough** and is coughing more than they usually do.
- The patient feels **extremely tired** and finds daily activities (such as walking up the stairs, getting out of bed, daily chores) difficult.
- The patient **is withdrawn and less responsive to the world around them**, they forget to go to the toilet when they need to and stop eating and drinking.
- The patient’s **muscles are sore**, particularly their shoulders and legs. The area feels sore to the touch and can prevent the patient from carrying out daily tasks without pain.
- The patient has an **underlying health condition** that makes them more likely to get severely ill from COVID-19. You do not need to consider the impact of this underlying health condition on the patient’s health, only that this condition makes the patient more likely to get severely ill from COVID-19.

**Description S5: High dependency unit (severe)**

- The patient has **COVID-19.**
- The patient is in a **high dependency unit** of a hospital, where patients are cared for more extensively than a general ward, but not to the point of intensive care.
- The patient is using **supplemental oxygen through a face mask**.
- The patient is **short of breath at rest**.
- The patient’s **heart rate is very high**.
- The patient has a **fever** – their body temperature is over 38°C. They feel hotter than usual (particularly on their chest and back), have chills and are shivery.
- The patient has a **dry cough** and is coughing more than they usually do.
- The patient feels **extremely tired** and finds daily activities (such as walking up the stairs, getting out of bed, daily chores) difficult.
- The patient is **withdrawn and less responsive to the world around them**, they forget to go to the toilet when they need to and stop eating and drinking.
- The patient’s **muscles are sore**, particularly their shoulders and legs. The area feels sore to the touch and can prevent the patient from carrying out daily tasks without pain.
- The patient has an **underlying health condition** that makes them more likely to get severely ill from COVID-19. You do not need to consider the impact of this underlying health condition on the patient’s health, only that this condition makes the patient more likely to get severely ill from COVID-19.

**Description S6: ICU (critical)**

- The patient has **COVID-19, they cannot breathe on their own and will die if not treated**.
- The patient is in an **intensive care unit** in a hospital.
- The patient has a **breathing tube inserted into their windpipe** to allow a machine to breathe for them.
- The patient is **unable to breathe on their own**.
- The patient has **multi-organ dysfunction/failure** of more than 1 of the following organ systems: respiratory (lungs), cardiovascular (heart and blood vessels), kidney, liver, and/or central nervous systems (brain and spinal cord) and requires organ support.
- The patient is **unconscious**.
- The patient has an underlying health condition that makes them more likely to get severely ill from COVID-19. You do not need to consider the impact of this underlying health condition on the patient’s health, only that this condition makes the patient more likely to get severely ill from COVID-19.

**Description S7: Recovered (no long-term sequelae)**

- The patient **has had** **COVID-19 and has now recovered without any long-term health issues**.
- The patient has an **underlying health condition** that makes them more likely to get severely ill from COVID-19. You do not need to consider the impact of this underlying health condition on the patient’s health, only that this condition makes the patient more likely to get severely ill from COVID-19.

**Description S8: Recovered (long-term sequelae)**

- The patient **has had COVID-19 and is now suffering from long-term health issues** as a result.
- The patient has an **underlying health condition** that makes them more likely to get severely ill from COVID-19. You do not need to consider the impact of this underlying health condition on the patient’s health, only that this condition makes the patient more likely to get severely ill from COVID-19.
- The patient feels **extremely tired** and finds daily activities (such as walking up the stairs, getting out of bed, daily chores) difficult.
- The patient is **short of breath** and feels as though they cannot get enough air in their lungs. They find it difficult to inhale and exhale. They have a tight chest.
- The patient’s **muscles and/or joints are sore**, particularly their shoulders and legs. The area feels sore to the touch and can prevent the patient from carrying out daily tasks without pain.

**Additional details on vignette development**

Given the considerable heterogeneity across the patient population, details relating to underlying conditions and risk factors experienced by patients were not reported in the vignettes. However, the vignettes did note that patients have an underlying health condition or risk factor that makes them more likely to become severely ill, given this knowledge may impact emotional wellbeing.

The most prevalent COVID-19 symptoms identified in the literature were used to inform the symptoms to be included in the vignettes. Prevalence data were sourced from an SLR conducted by MSD, which investigated the clinical, economic and humanistic burden of COVID-19, a COVID-19 Infection survey published by the Office for National Statistics (ONS), which reported the prevalence of COVID-19 symptoms in patients testing positive for the disease between 1st December 2020 to 30th April 2021 and the MOVe-OUT trial. No data were identified to inform the prevalence of symptoms according to COVID-19 severity, as defined in the MOVe-OUT trial. It was therefore assumed that the most common symptoms experienced in the wider COVID-19 population were representative of patients with mild to moderate disease who are treated in the outpatient setting. The most prevalent COVID-19 symptoms identified in the SLR were fever (between 85.4–83.6%), cough (between 68.7–62.7%) and fatigue (between 39.4–19.9%). The ONS survey reported the most prevalent symptoms to be cough (34.5%), fatigue (33.7%), headache (33.0%) and muscle pain (24.3%).([1](#_ENREF_1))Similarly, in the MOVe-OUT trial, the most prevalent self-reported signs and symptoms of COVID-19 at baseline were cough (81.7%), fatigue (76.1%), muscle or body aches (65.3%), headache (64.4%), nasal congestion (62%), feeling hot or feverish (54.1%) and loss of smell (45.9%).([2](#_ENREF_2)) Considering these three sources, the symptoms of fever, cough, fatigue, headache, muscle pain, loss of smell and nasal congestion were selected to represent the experience of a “typical” COVID-19 patient in the outpatient setting within the vignettes (S2 and S3).

No data were identified to inform the prevalence of symptoms for patients with severe disease as defined in the MOVe-OUT trial, and thus it was assumed that the most common symptoms experienced by patients requiring hospitalisation were representative of patients with severe disease. Symptom prevalence data for hospitalised patients were reported in a prospective observational study by The International Severe Acute Respiratory and Emerging Infection Consortium (ISARIC) and were used to inform the symptoms selected for the vignettes reflecting the hospitalised setting.([3](#_ENREF_3)) The study included 99,623 patients, with 79% of these being recruited from the UK. The most common symptoms identified were fever (68.7%), cough (68.3%), shortness of breath (65.8%), fatigue (46.4%), confusion (27.3%) and muscle pains (20.1%). These symptoms were therefore selected to represent the experience of a “typical” COVID-19 patient in the hospitalised setting (S4 and S5) (note: the disease severity concept of the vignettes already includes details relating to shortness of breath). Patients in the ICU ventilated (critical) vignette (S6) are described as being unconscious, and therefore would not be aware of any signs and symptoms they were experiencing. As such, no signs and symptoms were included in this vignette.

An ONS survey conducted in a sample of over 20,000 participants who tested positive for COVID-19 between 26th April 2020–6th March 2021 reported the prevalence of long COVID symptoms (persisting for more than four weeks after the first suspected COVID-19 episode). The most commonly reported symptoms were fatigue (85%), shortness of breath (63%), and muscle pain (48%).([4](#_ENREF_4)) These symptoms were therefore selected to inform the long-term complications experienced by patients recovered from COVID-19 in the relevant vignette (S8).

**References**

1. Office for National Statistics (ONS). Coronavirus (COVID-19) Infection Survey: England. <https://www.ons.gov.uk/peoplepopulationandcommunity/healthandsocialcare/conditionsanddiseases/datasets/coronaviruscovid19infectionsurveydata>. Accessed June 30, 2021. [

2. Guan Y, Puenpatom A, Johnson MG, Zhang Y, Zhao Y, Surber J, et al. Impact of Molnupiravir Treatment on Patient-Reported COVID-19 Symptoms in the Phase 3 MOVe-OUT Trial: A Randomized, Placebo-Controlled Trial. Clinical Infectious Diseases. 2023;77(11):1521-30.

3. Abdukahil SA, Abe R, Abel L, Absil L, Acker A, Adachi S, et al. COVID-19 symptoms at hospital admission vary with age and sex: results from the ISARIC prospective multinational observational study. Infection. 2021.

4. Office for National Statistics (ONS). Prevalence of ongoing symptoms following coronavirus (COVID-19) infection in the UK: 1 April 2021. <https://www.ons.gov.uk/peoplepopulationandcommunity/healthandsocialcare/conditionsanddiseases/bulletins/prevalenceofongoingsymptomsfollowingcoronaviruscovid19infectionintheuk/1april2021>. Accessed June 30, 2021. [
