# Supplementary material for: Health-related quality of life in COVID-19 in the United Kingdom: a vignette study
Source: Health Econ Rev. 2026 May 20;16:85. doi: 10.1186/s13561-026-00781-5 (PMC13366983; doi:10.1186/s13561-026-00781-5)
Supplement: Supplementary file 2 — Additional file 2. Subgroup analysis results. Presents a table of the EQ-5D-5L utility scores of vignettes by prior COVID-19 infection status, COVID-19 status of close friends or family, and vaccination status. [file 13561_2026_781_MOESM2_ESM.docx]

**Additional file 2**

Table 1: Summary of EQ-5D-5L utility scores of vignettes by prior COVID-19 infection status, COVID-19 status of close friends or family, and vaccination status

| **Variable** | **Parameter** | **Prior COVID-19 infection^a^** | | | **COVID-19 status of close friends or family^b^** | | | **Vaccination status^c^** | | | |
| --- | --- | --- | --- | --- | --- | --- | --- | --- | --- | --- | --- |
|  |  | **Yes (N=59)** | **No (N=440)** | **p-value*** | **Yes (N=339)** | **No (N=155)** | **p-value*** | **Fully vaccinated (N=419)** | **Partly vaccinated (N=27)** | **Not vaccinated (N=51)** | **p-value^+^** |
| S1 | Mean (SD) | 0.72 (0.23) | 0.73 (0.22) | 0.41 | 0.72 (0.22) | 0.75 (0.21) | 0.12 | 0.73 (0.22) | 0.71 (0.20) | 0.76 (0.23) | 0.36 |
|  | Median (IQR) | 0.72 (0.23) | 0.78 (0.22) |  | 0.75 (0.23) | 0.78 (0.22) |  | 0.78 (0.22) | 0.70 (0.24) | 0.81 (0.33) |  |
| S2 | Mean (SD) | 0.30 (0.26) | 0.29 (0.26) | 0.75 | 0.28 (0.26) | 0.31 (0.25) | 0.23 | 0.29 (0.26) | 0.32 (0.26) | 0.29 (0.24) | 0.84 |
|  | Median (IQR) | 0.30 (0.37) | 0.30 (0.42) |  | 0.29 (0.42) | 0.32 (0.34) |  | 0.31 (0.42) | 0.33 (0.38) | 0.28 (0.37) |  |
| S3 | Mean (SD) | 0.35 (0.27) | 0.31 (0.26) | 0.33 | 0.32 (0.27) | 0.31 (0.26) | 0.53 | 0.31 (0.27) | 0.38 (0.25) | 0.32 (0.26) | 0.46 |
|  | Median (IQR) | 0.42 (0.37) | 0.36 (0.38) |  | 0.40 (0.40) | 0.33 (0.36) |  | 0.37 (0.41) | 0.45 (0.31) | 0.31 (0.36) |  |
| S4 | Mean (SD) | -0.19 (0.28) | -0.18 (0.23) | 0.56 | -0.19 (0.24) | -0.16 (0.24) | 0.26 | -0.18 (0.23) | -0.17 (0.23) | -0.18 (0.28) | 0.60 |
|  | Median (IQR) | -0.22 (0.37) | -0.20 (0.26) |  | -0.22 (0.29) | -0.19 (0.26) |  | -0.20 (0.26) | -0.15 (0.26) | -0.22 (0.25) |  |
| S5 | Mean (SD) | -0.10 (0.25) | -0.12 (0.25) | 0.54 | -0.13 (0.23) | -0.09 (0.28) | 0.59 | -0.13 (0.25) | -0.06 (0.21) | -0.04 (0.28) | 0.06 |
|  | Median (IQR) | -0.14 (0.23) | -0.14 (0.31) |  | -0.15 (0.29) | -0.13 (0.33) |  | -0.15 (0.31) | -0.11 (0.30) | -0.11 (0.37) |  |
| S6 | Mean (SD) | -0.37 (0.17) | -0.38 (0.14) | 0.93 | -0.38 (0.14) | -0.37 (0.15) | 0.91 | -0.38 (0.14) | -0.39 (0.16) | -0.37 (0.15) | 0.89 |
|  | Median (IQR) | -0.41 (0.28) | -0.36 (0.28) |  | -0.36 (0.28) | -0.41 (0.28) |  | -0.36 (0.28) | -0.45 (0.26) | -0.38 (0.28) |  |
| S7 | Mean (SD) | 0.89 (0.11) | 0.87 (0.15) | 0.57 | 0.88 (0.12) | 0.85 (0.18) | 0.13 | 0.87 (0.15) | 0.90 (0.09) | 0.88 (0.13) | 0.64 |
|  | Median (IQR) | 0.86 (0.13) | 0.86 (0.18) |  | 0.86 (0.17) | 0.86 (0.21) |  | 0.86 (0.18) | 0.86 (0.12) | 0.86 (0.14) |  |
| S8 | Mean (SD) | 0.24 (0.27) | 0.21 (0.29) | 0.56 | 0.22 (0.29) | 0.19 (0.28) | 0.32 | 0.20 (0.29) | 0.33 (0.23) | 0.22 (0.27) | 0.11 |
|  | Median (IQR) | 0.23 (0.45) | 0.23 (0.53) |  | 0.24 (0.53) | 0.19 (0.46) |  | 0.23 (0.53) | 0.43 (0.29) | 0.23 (0.51) |  |

**Footnotes**: ^a^One participant reported 'Prefer not to say' and was removed from this analysis. ^b^Six participants reported 'Don't know' or 'Prefer not to say' and were removed from this analysis. ^c^Three participants reported 'Prefer not to say' and was removed from this analysis.
*from Wilcoxon rank sum test. ^+^from Kruskal-Wallis H test.
**Abbreviations**: IQR, interquartile range; N, total number of respondents; n, subset number of respondents; SD, standard deviation.
