# Supplementary material for: Health-related quality of life in COVID-19 in the United Kingdom: a vignette study
Source: Health Econ Rev. 2026 May 20;16:85. doi: 10.1186/s13561-026-00781-5 (PMC13366983; doi:10.1186/s13561-026-00781-5)
Supplement: Supplementary file 3 — Additional file 3. EQ-5D-5L responses by domain. Presents a table displaying the proportion of participants reporting each level of the EQ-5D-5L for each domain. [file 13561_2026_781_MOESM3_ESM.docx]

**Additional file 3**

Supplementary Table 2: Proportion of participants reporting each level of the EQ-5D-5L for each domain

| **Domain** | **Level** | **Disease state** | | | | | | | |
| --- | --- | --- | --- | --- | --- | --- | --- | --- | --- |
|  |  | **S1** | **S2** | **S3** | **S4** | **S5** | **S6** | **S7** | **S8** |
| Mobility | No problems | 77.0% | 5.2% | 1.6% | 0.2% | 0.0% | 0.0% | 89.2% | 0.0% |
|  | Slight problems | 11.6% | 19.4% | 22.4% | 2.2% | 2.2% | 0.0% | 8.6% | 10.6% |
|  | Moderate problems | 8.0% | 50.6% | 49.0% | 9.2% | 17.0% | 2.0% | 1.8% | 47.6% |
|  | Severe problems | 3.4% | 23.2% | 25.2% | 42.4% | 53.4% | 1.2% | 0.2% | 40.0% |
|  | Unable to walk about | 0.0% | 1.6% | 1.8% | 46.0% | 27.4% | 96.8% | 0.2% | 1.8% |
| Self-care | No problems | 78.4% | 7.4% | 6.8% | 0.0% | 0.2% | 0.0% | 93.2% | 2.6% |
|  | Slight problems | 15.2% | 33.8% | 32.6% | 1.8% | 3.4% | 0.0% | 5.2% | 24.8% |
|  | Moderate problems | 5.6% | 44.6% | 44.6% | 10.8% | 17.0% | 0.2% | 1.2% | 42.4% |
|  | Severe problems | 0.8% | 14.0% | 14.8% | 44.2% | 47.6% | 1.0% | 0.2% | 27.8% |
|  | Unable to wash or dress | 0.0% | 0.2% | 1.2% | 43.2% | 31.8% | 98.8% | 0.2% | 2.4% |
| Usual activities | No problems | 46.4% | 0.4% | 0.4% | 0.0% | 0.0% | 0.2% | 83.4% | 0.0% |
|  | Slight problems | 33.2% | 7.6% | 12.8% | 0.6% | 0.4% | 0.0% | 13.8% | 6.6% |
|  | Moderate problems | 14.4% | 37.8% | 42.2% | 5.2% | 8.8% | 0.0% | 2.0% | 41.2% |
|  | Severe problems | 5.0% | 43.2% | 36.6% | 28.4% | 36.8% | 0.6% | 0.6% | 41.4% |
|  | Unable to do usual activities | 1.0% | 11.0% | 8.0% | 65.8% | 54.0% | 99.2% | 0.2% | 10.8% |
| Pain/discomfort | No pain/discomfort | 49.2% | 0.2% | 0.2% | 0.0% | 0.0% | 25.4% | 82.2% | 0.0% |
|  | Slight pain/discomfort | 30.0% | 7.6% | 10.4% | 1.2% | 2.2% | 2.2% | 15.2% | 8.0% |
|  | Moderate pain/discomfort | 17.6% | 50.8% | 52.2% | 23.0% | 30.8% | 4.6% | 2.2% | 52.2% |
|  | Severe pain/discomfort | 3.2% | 37.6% | 35.0% | 49.6% | 52.6% | 9.6% | 0.2% | 34.0% |
|  | Extreme pain/discomfort | 0.0% | 3.8% | 2.2% | 26.2% | 14.4% | 58.2% | 0.2% | 5.8% |
| Anxiety/depression | Not anxious/depressed | 29.0% | 11.6% | 12.2% | 4.0% | 3.4% | 39.4% | 47.6% | 7.4% |
|  | Slightly anxious/depressed | 35.0% | 24.6% | 24.8% | 3.6% | 4.4% | 1.4% | 38.2% | 14.2% |
|  | Moderately anxious/depressed | 27.8% | 38.2% | 39.4% | 18.6% | 19.2% | 5.2% | 12.4% | 38.2% |
|  | Severely anxious/depressed | 6.2% | 18.8% | 18.2% | 40.4% | 42.8% | 8.0% | 1.0% | 28.2% |
|  | Extremely anxious/depressed | 2.0% | 6.8% | 5.4% | 33.4% | 30.2% | 46.0% | 0.8% | 12.0% |

**Footnotes:** The disease states were as follows: S1, Baseline (pre-infection); S2, Outpatient (mild); S3, Outpatient (moderate); S4, General hospital ward (severe); S5, High dependency unit (severe); S6, ICU (critical); S7, Recovered (no long-term sequelae); S8, Recovered (long-term sequelae). The grey highlighting indicates the level that was most commonly reported by participants for each domain.
